# Evaluating the feasibility and efficacy of a home-based combined high intensity interval and moderate intensity training program for increasing physical activity among low-active adults: A randomized pilot trial

**DOI:** 10.1371/journal.pone.0281985

**Published:** 2023-02-21

**Authors:** Beth A. Lewis, Katie Schuver, Shira Dunsiger

**Affiliations:** 1 School of Kinesiology, University of Minnesota, Minneapolis, Minnesota, United States of America; 2 Center for Health Promotion and Health Equity, Brown University, Providence, Rhode Island, United States of America; Imam Abdulrahman Bin Faisal University, SAUDI ARABIA

## Abstract

**Background:**

High intensity interval training (HIIT), which includes short bursts of high-intensity physical activity (PA) followed by recovery, can increase PA by addressing time barriers and improving PA enjoyment. The purpose of this pilot study was to examine the feasibility and preliminary efficacy of a home-based HIIT intervention on PA.

**Methods:**

Low active adults (n = 47) were randomly assigned to a home-based HIIT intervention or wait-list control lasting 12 weeks. Participants in the HIIT intervention received motivational phone sessions based on Self-Determination Theory and accessed a website that included workout instructions and videos demonstrating proper form.

**Results:**

The HIIT intervention appears feasible based on retention, recruitment, adherence to the counseling sessions, follow-up rates, and the consumer satisfaction survey. HIIT participants reported more minutes of vigorous intensity PA at six weeks relative to control (no differences at 12 weeks). HIIT participants reported higher levels of self-efficacy for PA, enjoyment of PA, outcome expectations related to PA, and positive engagement with PA than the control.

**Conclusions:**

This study provides evidence for feasibility and possible efficacy of a home-based HIIT intervention for vigorous intensity PA; however, additional studies are needed with larger samples sizes to confirm efficacy of home-based HIIT interventions.

**Trial registration:**

Clinical Trials Number: NCT03479177.

## Introduction

Physical activity (PA) is associated with numerous health benefits including the reduced risk of cardiovascular disease, type 2 diabetes, stroke, depression, anxiety, and sleep problems [1]. It is also associated with improved bone health [2] and cognitive functioning [3]. Consequently, the US Department of Health and Human Services recommend that adults engage in 75–150 minutes of vigorous intensity PA per week or 150–300 minutes of moderate intensity PA per week, and muscle strengthening activities of moderate or greater intensity for two or more days per week [1]. Unfortunately, only 19% of women and 26% of men meet the aerobic and muscle strengthening PA guidelines.

Research indicates that vigorous intensity PA may be superior to moderate intensity PA for reducing the risk of chronic diseases such as type 2 diabetes and cardiovascular disease [4, 5]. Specifically, one study found that the CVD mortality hazard ratios were more than two times higher among obese individuals who met the moderate intensity guidelines vs. those who met the vigorous intensity guidelines. Additionally, research indicates that muscle strengthening activities may be particularly important for reducing chronic disease risk [6].

Behavioral interventions are efficacious for increasing PA among low-active and sedentary adults [7]. However, long-term adherence is problematic. Additionally, most behavioral interventions encourage moderate intensity PA but do not target vigorous intensity or muscle strengthening activities [7]. This is a significant limitation given the numerous health benefits related to vigorous intensity and muscle strengthening activities [8].

Recent research has examined the efficacy of High Intensity Interval Training (HIIT) as a strategy to address the limitations of previous traditional PA studies [9–11]. High Intensity Interval Training (HIIT) consists of short bouts of vigorous intensity PA followed by rest or active recovery [12]. HIIT addresses the “lack of time” barrier since it takes less time than moderate intensity PA [13]. Additionally, HIIT is advantageous to traditional vigorous intensity activity since it includes active recovery or rest. Some studies indicate that HIIT may be more enjoyable than moderate intensity, continuous PA [14, 15]; however, others have argued that this is not the case [16]. Biddle and Batterham [17] summarized the arguments for and against promoting HIIT as a viable PA program. Biddle argued that due to the aversive nature of high intensity exercise, HIIT is not a viable public health strategy. However, Batterham argued that this idea is outdated and based on traditional continuous vigorous intensity program rather than interval training. In sum, more research is needed to better understand the viability of HIIT and the effect of HIIT on affective responses (i.e., pleasurable vs. unpleasurable) to PA.

A majority of HIIT studies have been conducted in a laboratory, which does not address long-term adherence to PA nor do lab-based studies generalize to real world settings [8]. Studies that have examined home-based HIIT have yielded in mixed results [11, 18, 19]. Major limitations for many of these studies is the lack of focus on strengthening activities, which is inconsistent with PA guidelines, and no or little focus on theory-based behavioral strategies to increase adherence.

The purpose of this pilot study was to examine the feasibility and efficacy of a 12-week home-based High Intensity Interval Training (HIIT) program among low-active individuals relative to a wait-list control arm. Self-Determination Theory informed the intervention for this study, which addressed limitations of the previous home-based studies by including muscle strengthening exercises and a motivational component. As outlined by Bowen et al., [20], feasibility studies should examine acceptability and implementation, and conduct limited efficacy testing. For this study, acceptability will be achieved if participants attend a mean number of 75% of the motivational sessions and rate the intervention a mean number of five on a six-point Likert scale. Implementation will be achieved if we recruit at least 40 participants over six months and retain at least 90% of the sample at 12 weeks. We determined that 40 participants would be adequate to determine if we could sustain a recruitment rate of 6–7 participants per month, which could inform a future trial seeking to recruit 200 participants over 2.5 years.

Regarding preliminary efficacy, we hypothesized that participants randomized to the HIIT intervention would exhibit more moderate to vigorous intensity (MVPA) and vigorous intensity PA minutes per week at 6 and 12 weeks than those randomized to the wait-list control. We also hypothesized that the HIIT intervention group would report greater increases in PA-related psychosocial variables including social support, self-efficacy, enjoyment, and outcome expectations than the wait-list control arm. Affective responses to the exercise sessions were also examined.

## Methods

### Overview of study

This study was a randomized controlled single blind pilot study in which participant were recruited from August, 2018 to January, 2019 (assessments were completed by April, 2019). Participants (n = 47) were randomized 1:1 to either a home-based High Intensity Interval Training program (HIIT) or a wait-list control arm each lasting 12 weeks. Participants also completed a moderate intensity session one time per week in order to compare affective responses to the HIIT sessions. The primary dependent variable was PA minutes per week at six and 12 weeks based on a modified version of the 7-Day Physical Activity Recall. Secondary dependent variables included social support, self-efficacy, enjoyment, outcome expectancies, and affective responses to PA. Participants completed an online written informed consent form prior to participating. This study was approved by the University of Minnesota’s Institutional Review Board, which reports to the Office of the Vice President for Research. The wait-list condition was given the opportunity to complete the HIIT intervention following their 12-week assessment.

### Participants

Low active participants ages 18 years of age or older were recruited from the northern region of the United States through worksite emails. Eligibility criteria were assessed using a telephone-based interview. Exclusion criteria included the following: (1) Engaging in PA for more than 90 minutes each week; (2) lack of access to the Internet; (3) history of coronary heart disease (history of myocardial infarction, symptoms of angina); (4) orthopedic problems that would limit physical activity participation; (5) diabetes, stroke, osteoarthritis, and any other medical condition that may make physical activity unsafe or unwise; (6) current or planned pregnancy; (7) psychiatric hospitalization within the last six months; (8) psychosis or current suicidal ideation; and (9) unwilling to be randomized to either of the study conditions.

### Measures

#### Primary aim: Feasibility

Feasibility will be assessed by examining acceptability and implementation. Level of acceptability will be based on attendance at the motivational phone sessions and the consumer satisfaction questionnaire (items were on a seven point Likert scale). Implementation will be based on recruitment and retention rates. Additionally, as recommended by Eldridge et al. [21], we will examine several variables to answer the question, “Can this study be done?” Specifically, we will examine the number of eligible participants, percent of participants randomized from the eligible participant pool, follow-up rates, and time needed to collect data.

#### Secondary dependent variables: PA minutes per week and psychosocial variables

PA minutes per week was assessed at baseline, six and 12 weeks based on a modified version of the 7-Day Physical Activity Recall Interview (PAR). The PAR is considered the gold standard for assessing PA via self-report [22, 23]. Specifically, participants self-reported their PA on an online questionnaire that was based on the PAR. The ActiGraph GT9X-BT, which is an accelerometer worn at the hip that objectively measures PA, was used to objectively measure PA. However, data from the ActiGraph was not used for the current study due to recent evidence suggesting that accelerometers are not valid for assessing HIIT sessions [24].

Social support for engaging in PA was measured using the 13-item Social Support for Physical Activity Scale [25]. The internal consistency for this scale was .90 for family and .84 for friends. Validity has been established in that it has been shown to correlate with vigorous activity [25]. Self-efficacy was examined using a 5-item scale that examines one’s confidence to engage in PA in different situations [26]. The internal consistency of this measure was .85 and it has good test-retest reliability [26]. The 18-item Physical Activity Enjoyment Scale (PACES) was used to assess PA enjoyment [27]. The internal consistency was .91 and validity has been demonstrated [27]. The 19-item Outcome Expectations for Exercise Scale was used to assess the participant’s expectation that PA will lead to a particular outcome and how much the participant values that outcome [28]. Internal consistency was .91 and validity has been established [28]. The 20-item Multi-Dimensional Fatigue Inventory assessed several areas related to fatigue including: General fatigue, physical fatigue, mental fatigue, reduced activity, and reduced motivation. Internal consistency was .80 and there is adequate convergent validity with other similar scales [29]. Finally, the 12-item Exercise-Induced Feeling Inventory assessed four feeling states including tranquility, revitalization, physical exhaustion, and positive engagement. The measure has been shown to correlate with related constructs [30].

The Feeling Scale (FS) was administered to the HIIT intervention participants to assess affective responses to the HIIT and moderate intensity sessions [31]. Specifically, one time per week, participants rated their affect and enjoyment following one HIIT and one moderate intensity session. The items were, “How do you currently feel?” (11-point Likert scale ranging from “very bad” to “very good”) and “Use the following scale to indicate how much you will enjoy (or enjoyed) this exercise session (7-point Likert scale ranging from “not all” to “extremely”).” The FS correlates with the rate of perceived exertion scale (RPE) [31].

### Procedure

In response to the worksite emails, potential participants called, emailed, or texted a study line. A telephone screening interview, which was adapted from the 10-item Physical Activity Readiness Questionnaire [32, 33], determined eligibility for the study. The intervention coordinator gave detailed information about the study and the consent form was reviewed. If still interested, participants were next emailed the consent form and online questionnaires. Upon completion of the online consent form and questionnaires, the ActiGraph was mailed to the participants to be worn for seven days. Once the ActiGraph was returned, the research assistant called or texted the participant to schedule the randomization session. Participants were randomly assigned over the telephone via a 1:1 ratio to either the HIIT intervention or wait-list control. The random numbers table was generated using Microsoft excel by the project coordinator who did not conduct the assessments. The wait-list control condition had the option of receiving the HIIT intervention after 12 weeks.

#### HIIT intervention

The HIIT intervention was a 12-week high intensity interval training workout that consisted of home-based HIIT sessions prescribed by the telephone counselor. The content of the HIIT sessions were determined based on exercises the participant could confidently engage in (e.g., regular push-ups vs. knee push-ups vs. wall push-ups). Participants in the HIIT condition were told when PA should be stopped for safety reasons in order to minimize risk to the participant. The goal was to engage in four PA sessions per week including three HIIT sessions and one moderate intensity continuous session. The moderate intensity session was included to allow for a comparison of affective responses during the HIIT and moderate intensity sessions. Ten percent of the HIIT sessions were audiotaped and reviewed by the PhD-level project director to ensure fidelity to the telephone sessions. The project director completed protocol checklists when listening to the sessions.

The HIIT intervention included telephone calls delivered by a master’s level health coach who is a trained personal trainer and holds an American College of Sports Medicine Certified Exercise Physiologists^®^ (ACSM-EP^®^) credential. Participants received telephone calls weekly during the first month and bi-weekly during months two and three for a total of eight calls. The sessions were based on Self-Determination Theory (SDT), which postulates that intrinsically motivated individuals are more likely to increase PA than those who are extrinsically motivated [34, 35]. Intrinsic motivation is increased when individuals have a choice for type of PA and they are allowed to self-direct. Therefore, participants were allowed to choose between the different HIIT interventions. Also, consistent with SDT, the counselor worked with the participant to increase enjoyment of each session. The intervention component and targeted psychosocial construct is summarized in Table 1. Overall, the counselor and participant worked to collaborate on how to make PA a part of the participant’s self-identify and how to fit PA into their daily routine. The counseling sessions were recorded for quality control purposes by the research staff.

**Table 1:** Summary of intervention component targeting psychosocial variables.

Psychosocial Variable	Description	Explanation of How Intervention Targeted the Variable	Behavior Change Technique
Autonomy	Feeling in control of your actions can be helpful for increasing PA.	Participants were provided a variety of workouts and given the autonomy to choose which to participant in and when.	Prompt specific goal setting; prompt review of behavioral goals; prompt self-monitoring of behavior; time management.
Competence	Mastering and learning new skills can lead to better PA adherence.	Participants learned new strength training skills as part of the various workouts that were available. The counselor worked with the participant to think about how much they were accomplishing (e.g., new skill, knee push-up vs. wall push-up, faster completion rate).	Provide instruction; provide general encouragement.
Social Support/Relatedness	Social support from others either through encouragement or engaging in PA physical activity together can be helpful for PA adherence.	Participants were encouraged to elicit support from friends and family and discuss their PA plans with their support network. Counselors encouraged participants to reach out to others to exercise with if this was motivating for the participant. The counselors also offered positive feedback for reaching goals.	Plan social support or social change; provide feedback on performance.
Enjoyment	Participants who report that they enjoy PA are more likely to engage in PA.	Participants were encouraged to identify positive feelings, associations, and experiences of enjoyment from regular PA. If participants do not typically enjoy PA in the moment, the counselor focused on positive feelings related to completing a PA session.	Provide information on consequences.
Outcome Expectations	Participants who expect positive outcomes as a result of PA will be more likely to engage in PA.	Participants were encouraged to identify benefits of PA; counselors presented information about regular PA and the consequences of action/inaction.	Provide information about behavior-health link.
Exercise-Induced Feeling	Experiencing positive feelings associated with PA including tranquility, revitalization, and positive engagement can be helpful for PA adherence.	Participants were encouraged to identify positive feeling states associated with PA; counselors discussed reframing strategies to address negative feelings and associations with exercise.	Prompt self-talk.
Self-Efficacy	Confidence to engage in PA in different situations can lead to higher levels of PA.	Participants encouraged to identify barriers to regular PA and counselors discussed possible strategies to overcome identified barriers.	Prompt barrier identification.

Note: Behavior Change Techniques from Abraham & Michie [39].

Sessions were approximately 30 minutes total including a five minute warm-up, 20 minutes of HIIT, and a five minute cool-down. The sessions were progressively overloaded using bodyweight. For example, a participant may have started with pushups on their knees, which progressed to pushups on their toes, overall repetitions of burpees were increased, and/or sprints improved from 45 to 40 seconds. Resistance training included bodyweight exercises such as squats, push-ups, lunges, and planks. Aerobic exercise included exercises such as jumping jacks, sprints, jump rope, high knees, and mountain climbers. Resistance exercises aimed to increase strength and stability along with increasing heart rate and aerobic exercise aimed to increase heart rate. The session intensity was determined by RPE.

Participants also accessed a study website that included descriptions of several different HIIT sessions. The website also included videos that demonstrated the HIIT exercises. Examples of exercises included push-ups, squat jumps, walking lunges, mountain climbers, hill sprints, plank, and jumping rope. Participants chose the type of moderate intensity PA they participated in each week. The goal was for participants to reach 70–85% of their maximum heart rate for the HIIT session with the high intensity components reaching 80–90%. The goal for the moderate sessions were 55–70% of maximum heart rate.

### Data analysis

Descriptive statistics were used to summarize baseline variables in the aggregate sample and t-tests and chi-squared tests were used to compare groups at baseline (as appropriate). Unadjusted means were reported over time by condition for each of the primary and secondary study outcomes.

A series of generalized linear models were used to examine intervention effects on absolute scores at follow-up (six and 12 weeks in a single model) controlling for baseline. Models included effects of time, condition, time* condition, and baseline value of the outcome. Fixed effects were estimated in each model. Repeated measures ANOVA was used to examine within subject differences on affective responses between the HIIT and moderate intensity PA sessions. All analyses were run in SAS 9.3 based on the intent to treat sample (all randomized participants included in the analysis).

## Results

Demographic information is summarized in Table 2. A majority of participants were female and the mean age was 34 (Range: 20–64). There were no differences between the study arms on the demographic variables.

**Table 2:** Baseline characteristics by study arms.

Variable	Total sample	HIIT	Control	P-value
	(n = 47)	(n = 23)	(n = 24)	
Age (mean in years)	34.26(10.86)	33.61(11.13)	34.88(10.78)	.694
Female	87.2%	91.3%	83.3%	.424
Race (%)				.196
Caucasian	61.7%	52.2%	70.8%	
Asian	12.8%	13.0%	12.5%	
African-American	10.6%	13.0%	8.3%	
Other	10.6%	13.0%	8.3%	
Refused	4.3%	8.7%	0.0%	
Hispanic (%)	10.6%	13.0%	8.3%	.610
Marital Status (% Married)	46.8%	47.8%	45.8%	.894
Education (% College Grad)	85.1%	91.3%	79.1%	.252
Income (% over $50,000)	42.6%	39.1%	45.8%	.651

Note: Age is reported as means with standard deviations in parentheses.

### Feasibility

**Fig 1:**
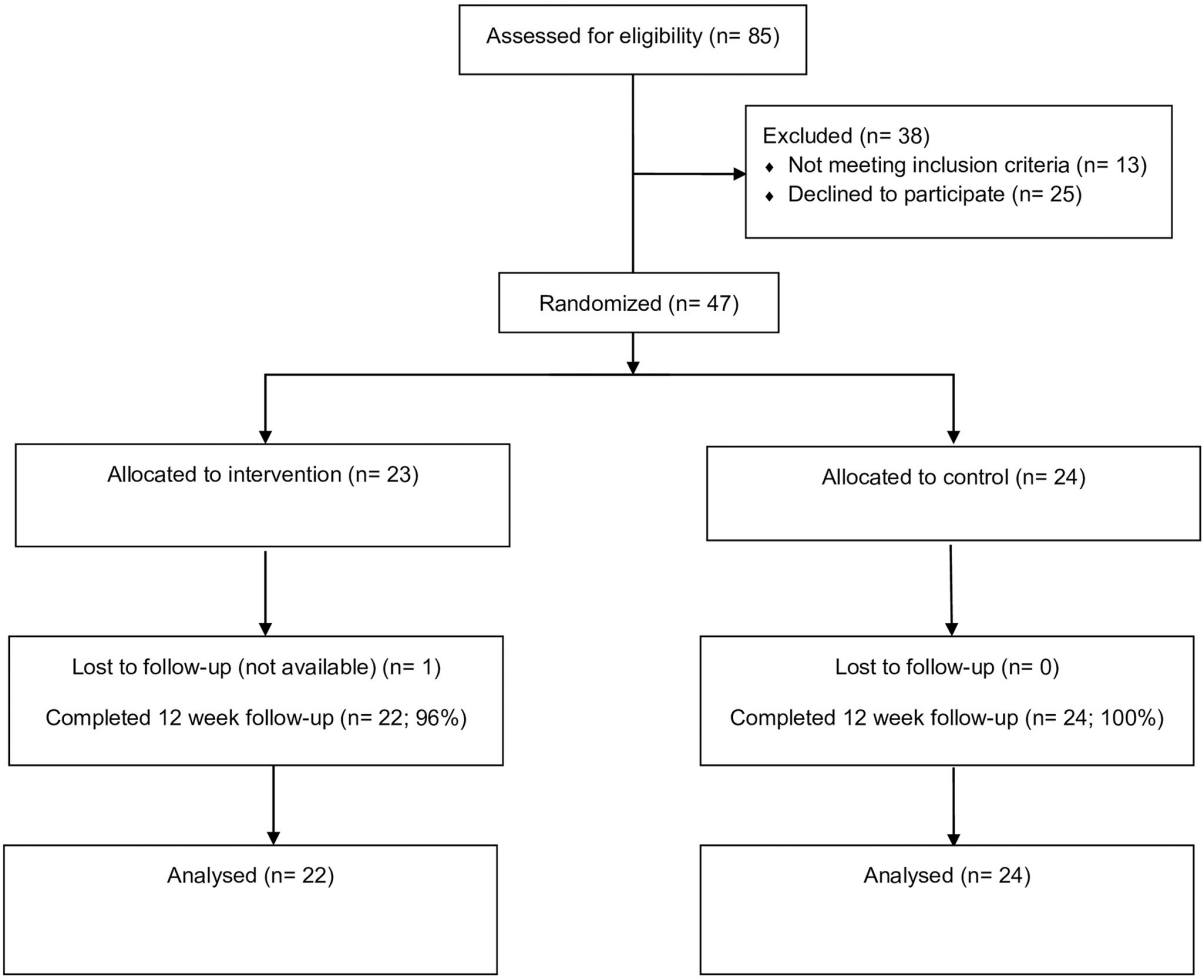
HIIT flow chart.

Recruitment is summarized in Fig 1. Among the 85 participants who completed the telephone screening interview, 72 were eligible. Sixty-five percent (n = 47) of the eligible participants were randomized over a five-month period of time. The retention rate was 98% for the HIIT intervention and 100% for the control condition at 12 weeks (retention was defined as completion of the 12-week questionnaires). Participants completed a mean number of 7.3, with a standard deviation (SD) of 0.83, of the eight telephone-based sessions (90%). The mean minutes for the telephone sessions was 12 minutes (SD = 2.00). The consumer satisfaction data is summarized in Table 3 (mean overall rating across the seven items was 5.07).

**Table 3:** Consumer satisfaction survey.

Variable	Mean (SD)
Overall Program	5.50 (1.57)
Website	5.00 (1.41)
Exercise Videos	4.82 (1.78)
Exercise Information from the Website	4.91 (1.58)
Telephonic exercise sessions	5.73 (1.68)
Information from the Exercise Counselor	6.42 (1.17)
Online Exercise Logs	3.08 (1.88)

Note: Items were on a seven point Likert scale.

### Physical activity

Unadjusted PA minutes per week by study arm are reported in Table 4. There were no differences between groups for moderate to vigorous intensity PA (p’s>.05) in the adjusted model across time (p-value for the treatment*time = .25). However, the HIIT condition reported a greater number of vigorous intensity PA minutes per week at six weeks (mean = 40.00, SD = 43.62) when compared to the wait-list condition (mean = 16.25, SD = 35.64, d = .59), *f* (1, 44) = 5.15, *p* = .04. There were no differences for vigorous intensity PA at 12 weeks (means for HIIT and Control were 61.83, SD = 77.78 and 34.58, SD = 76.44 respectively, d = .35).

**Table 4:** The effect of the HIIT on physical activity: Unadjusted effects.

Variable	HIIT (n = 23)			Control (n = 24)		
	Baseline	6 Weeks	12 Weeks	Baseline	6 Weeks	12 Weeks
Total MVPA min/week	53.26 (89.5)	98.26 (98.3)	113.91 (84.7)	111.88 (101.5)	80.42 (106.2)	127.29 (162.6)
Vigorous Only min/week	3.91 (18.8)	40.00 (43.6)	61.83 (77.8)	14.17 (29.5)	16.25 (35.6)	34.58 (76.4)

Abbreviations: MVPA = Moderate to Vigorous Intensity Physical Activity. The above values are means. Standard deviations are in parentheses.

### Psychosocial variables

The effect of the HIIT intervention on several psychosocial variables is summarized in Table 5. There was no effect of the HIIT intervention on social support from family, social support from friends, overall fatigue, physical fatigue, mental fatigue, feelings of revitalization, and tranquility relative to the control group. Participants in the HIIT intervention reported higher levels of self-efficacy for physical activity, (b = .56, SE = .16, p = .001, d = .67), enjoyment, (b = 6.84, SE = 3.27, p = .039, d = .59), outcome expectations, (b = 3.73, SE = 1.83, p = .048, d = .45), and positive engagement, (b = .99, SE = .52, p = .045, d = .66) relative to the control after adjusting for baseline values.

**Table 5:** The effect of HIIT on psychosocial variables: Unadjusted effects.

Variable	HIIT (n = 23)		Control (n = 24)	
	Baseline	12 Weeks	Baseline	12 Weeks
Physical Activity Self-Efficacy	2.52(.95)	2.75(.97)	2.34(1.04)	2.08(1.00)
Family Participation Social Support	23.61(10.67)	25.26(13.55)	28.38(12.90)	25.30(14.66)
Family Rewards and Punishment	5.17(5.13)	3.65(2.52)	5.54(5.01)	5.08(4.28)
Friend Participation Social Support	19.17(8.24)	19.79(9.72)	20.21(9.33)	22.59(15.26)
Physical Activity Enjoyment Scale	91.00(19.11)	99.00(20.87)	91.30(19.92)	86.80(20.16)
Outcome Expectations for Exercise	36.48(7.40)	38.65(5.80)	37.38(5.50)	35.96(6.12)
Multidimensional Fatigue Inventory	59.00(10.83)	47.74(14.70)	58.17(9.06)	55.96(11.01)
EFI: Positive Engagement Subscale	8.83(2.89)	10.48(2.87)	8.88(2.58)	8.71(2.49)
EFI: Revitalization Subscale	6.09(2.21)	9.57(3.29)	6.92(2.83)	7.54(3.27)
EFI: Tranquility Subscale	9.83(3.23)	10.96(2.71)	9.83(3.10)	9.75(3.01)
EFI: Physical Exhaustion Subscale	9.30(2.75)	6.65(3.13)	9.04(3.24)	8.25(3.57)

Note: EFI = Exercise Feeling Inventory. The above values are means (standard deviations in parentheses).

### Affective responses to moderate vs. HIIT sessions

Sixty-five percent of the participants completed at least one moderate intensity session affect log and 83% completed at least one HIIT log during the intervention. Participants completed a mean number of 4.26 (SD = 4.65) moderate intensity logs and 6.09 (SD = 6.84) HIIT logs. Among the participants randomly assigned to the HIIT intervention, there were no differences between the moderate and HIIT sessions regarding affective responses (i.e., pleasure and enjoyment; p’s > .05).

## Discussion

The home-based HIIT intervention appears feasible based on meeting the acceptability and implementation parameters. Specifically, we met our recruitment goal within the intended timeframe, which was to recruit 40 participants in six months. Our retention rate (98%) was higher than our 90% retention goal. We randomized two-thirds of the eligible participants. Additionally, participants attended a mean number of 90% of the motivational sessions, which exceeded our 75% goal. The mean rating across the seven consumer satisfaction items was 5.07, which exceed our goal of five. Participants rated the information from the exercise counselor and the exercise sessions as the highest and the online exercise logs the lowest.

Regarding efficacy, contrary to our hypotheses and previous studies [10], participants randomly assigned to the HIIT intervention did not report higher levels of overall PA than those randomly assigned to the control condition. This is potentially due to the control arm surprisingly increasing their levels of PA despite not receiving an intervention. Perhaps participants who enrolled in the study were motivated to increase their PA prior to randomization and therefore, increased their PA levels despite not receiving an intervention. Consistent with our hypotheses, participants in the HIIT intervention reported greater increases in vigorous intensity PA than those in the control at six weeks.

The study was not powered to examine which psychosocial variables mediated the effect of the intervention on PA. Therefore, in order to inform future trials examining mediation, only the effect of the intervention on the psychosocial variables was examined. Self-efficacy of physical activity, enjoyment, outcome expectations (i.e., expected outcomes and value of those outcomes as a result of PA), and positive engagement (i.e., upbeat, enthusiastic, happy) increased from baseline to 12 weeks for the intervention relative to the control. This is consistent with previous studies indicating that these psychosocial variables are related to physical activity behavior change [36].

Among the participants randomized to the HIIT sessions, there were no differences between the moderate and HIIT sessions regarding affective responses (i.e., ratings of pleasure and enjoyment). This is inconsistent with previous HIIT studies [14, 15] that have found that HIIT is more enjoyable than moderate intensity. The lack of differences is also inconsistent with research arguing that vigorous intensity PA elicits a lower level of pleasure than moderate intensity PA [16]. The findings in the present study are limited by the low number of logs completed and the inconsistency between compliance with completing the moderate vs. HIIT logs. Additional research is needed to better understand affective responses to HIIT vs. moderate intensity sessions.

There were several strengths to this study. First, the examination of a home-based HIIT intervention is novel given a majority of HIIT studies have been lab-based. Second, the study was strong methodologically in that participants were randomized to the conditions, there was quality control for the counseling sessions, an experienced health coach delivered the intervention, and validated measures were used. Finally, the study sample was relatively diverse when compared to the overall population of the recruitment area. Despite these strengths, there were some limitations. First, data from the objective measure of PA was not included given the lack of validity for using accelerometers to assess HIIT sessions [24]. Second, the PAR, which is typically administered via an interview, was modified and administered as a questionnaire. Third, the intervention was short in duration (i.e., 12 weeks). Fourth, only 13% of the sample included males, which decreases the generalizability of the sample. Finally, satisfaction and autonomous motivation were not assessed even through these variables are an integral part of Social Determination Theory.

The HIIT intervention appears feasible based on retention, recruitment, adherence to the counseling sessions, follow-up rates, and the consumer satisfaction survey. Participants indicated they were the least satisfied with the online exercise logs suggesting that additional research is needed to improve upon the usability and acceptability of online exercise logs. Participants were the most satisfied with the exercise counseling sessions indicating that personal motivational sessions may be important for adherence to HIIT. Based on the feasibility data and the preliminary efficacy data indicating higher rates for vigorous intensity PA among the HIIT intervention relative to control, it appears that the HIIT intervention should be examined in a large trial. It will be important to examine affective responses to better understand how these responses influence adherence. Studies should explore integrating HIIT interventions within existing opportunities (e.g., physical education) and increase physical literacy [37]. Finally, there is a strong need to validate objective monitors of PA for HIIT sessions and develop psychosocial measures specifically developed for HIIT interventions such as the High-Intensity Interval Training Self-Efficacy Questionnaire (HIIT-SQ) [38].

In summary, this small pilot study indicates that home-based HIIT is acceptable and feasible for low active adults. However, selection bias may have played a role in the acceptability of vigorous intensity PA. It is possible that participants who enrolled in the study were more open to vigorous intensity PA than those not interested in the study. HIIT may be advantageous to traditional PA given it takes less time to meet the PA recommendation and it may be more enjoyable than moderate intensity activity. Future studies with larger sample sizes and objective measures of PA are needed to better understand if this study is viable for dissemination to low active adults.

## Supporting information

S1 Checklist CONSORT 2010 checklist of information to include when reporting a randomised trial*. (DOC)

S1 Data (XLSX)

S1 File (DOCX)
