# Supplementary material for: Evaluating the feasibility and efficacy of a home-based combined high intensity interval and moderate intensity training program for increasing physical activity among low-active adults: A randomized pilot trial
Source: PLoS One. 2023 Feb 21;18(2):e0281985. doi: 10.1371/journal.pone.0281985 (PMC9942957; doi:10.1371/journal.pone.0281985)
Supplement: S1 File — (DOCX) [file pone.0281985.s003.docx]

**PROTOCOL TITLE:**

The Effect of a Home-Based HIIT Intervention on Increasing Physical Activity among Low Active Adults

**PRINCIPAL INVESTIGATOR or FACULTY ADVISOR:**

Beth A Lewis
School of Kinesiology

612-625-0756

[blewis@umn.edu](mailto:blewis@umn.edu)

**STUDENT INVESTIGATOR:**

NA

**VERSION NUMBER/DATE:**

Version #3. 5/22/2018

**REVISION HISTORY**

| **Revision #** | **Version Date** | **Summary of Changes** | **Consent Change?** |
| --- | --- | --- | --- |
| 1 | March 15, 2018 | Addition of clinicaltrials.gov registration number and instruction in case of injury- added to consent form. | Yes. |
| 2 | 5/22/2018 | Update to phone screen, consent form with addition of 6 week assessment.  Addition of two questionnaires- PAR & Exercise Log  Adding research personnel- Laura Polikowsky & Cecilia Rosales | Yes. |
|  |  |  |  |
|  |  |  |  |
|  |  |  |  |
|  |  |  |  |

Table of Contents

[1.0 Objectives 6](#_Toc494449131)

[2.0 Background 6](#_Toc494449132)

[3.0 Study Endpoints/Events/Outcomes 6](#_Toc494449133)

[4.0 Study Intervention(s)/Investigational Agent(s) 6](#_Toc494449134)

[5.0 Procedures Involved 7](#_Toc494449135)

[6.0 Data and Specimen Banking 10](#_Toc494449136)

[7.0 Sharing of Results with Participants 10](#_Toc494449137)

[8.0 Study Population 9](#_Toc494449138)

[9.0 Vulnerable Populations 9](#_Toc494449139)

[10.0 Local Number of Participants 10](#_Toc494449140)

[11.0 Local Recruitment Methods 10](#_Toc494449141)

[12.0 Withdrawal of Participants 11](#_Toc494449142)

[13.0 Risks to Participants 11](#_Toc494449143)

[14.0 Potential Benefits to Participants 11](#_Toc494449144)

[15.0 Statistical Considerations 12](#_Toc494449145)

[16.0 Confidentiality 12](#_Toc494449146)

[17.0 Provisions to Monitor the Data to Ensure the Safety of Participants 12](#_Toc494449147)

[18.0 Provisions to Protect the Privacy Interests of Participants 13](#_Toc494449148)

[19.0 Compensation for Research-Related Injury 13](#_Toc494449149)

[20.0 Consent Process 14](#_Toc494449150)

[21.0 Setting 15](#_Toc494449151)

[22.0 Multi-Site Research 14](#_Toc494449152)

[23.0 Resources Available 15](#_Toc494449153)

[24.0 References 15](#_Toc494449154)

**ABBREVIATIONS/DEFINITIONS**

- NA

**STUDY SUMMARY**

| **Study Title** | The Effect of a Home-Based HIIT Intervention on Increasing Exercise among Low Active Adults |
| --- | --- |
| **Study Design** | The purpose of this study is to examine the effect of a home-based HIIT intervention on exercise among low active adults (defined as engaging in exercise 90 minutes or less per week). Participants will be randomly assigned to a HIIT-based intervention or a wait-list control each lasting 12 weeks (participants in the wait-list control condition will have the option of receiving the HIIT intervention following the 12 weeks). |
| **Primary Objective** | To examine the effect of a HIIT intervention on exercise minutes per week. |
| **Secondary Objective(s)** | To examine the effect of a HIIT intervention on weight and key psychosocial variables related to exercise. |
| **Research Intervention(s)/Investigational Agents** | HIIT is defined as an exercise protocol that intersperses a short period of high-intensity exercise with short periods of rest. The HIIT intervention will be designed to increase high intensity activity (70-85% of maximum heart rate) and will focus on engaging in exercises based on the participant’s current fitness and strength level. Even though high intensity is the goal, the participant will take frequent breaks throughout the exercise session to offset the high intensity nature of the exercises and potentially increase enjoyment of the exercise session. The participant will participate in eight telephone sessions over the 12 weeks that will instruct the participants on the home-based sessions and to motivate the participant to adhere to the exercise program. |
| **Scientific Assessment** | Not required, Minimial Risk Study |
| **IND/IDE # (if applicable)** | N/A |
| **IND/IDE Holder** | N/A |
| **Investigational Drug Services # (if applicable)** | N/A |
| **Study Population** | The target population will be individuals who exercise 90 minutes or less each week and do not have any health conditions that would limit exercise. |
| **Local Sample Size (number of participants recruited locally)** | 50 |

# Objectives

- 1. The purpose of this study is to examine the effect of a home-based HIIT intervention on exercise among low active adults (defined as engaging in physical activity 90 minutes or less per week). Participants will be randomly assigned to a HIIT-based intervention or a wait-list control each lasting 12 weeks (participants in the wait-list control condition will have the option of receiving the HIIT intervention following the 12 weeks).

# Background

- 1. Significance of Research Question/Purpose: A majority of previous studies have examined the effect of moderate-intensity exercise; however, few studies have examined the effect of home-based high intensity interval training on exercise adherence among low active individuals.
  2. Preliminary Data: N/A
  3. Existing Literature: High Intensity Interval Training (HIIT) consists of short repeated periods of intense effort (approximately 80-90% of an individual’s maximum heart rate) with recovery intervals in-between. HITT requires just a fraction of the time that is required for moderate intensity training (MIT; approximately 10-20% of the time commitment), and, therefore, addresses the “lack of time” barrier (Burgomaster et al., 2008; Gibala et al., 2006; Gibala, Little, Macdonald, & Hawley, 2012). Researchers have found that HITT leads to comparable or superior improvements in cardiometabolic health outcomes when compared to moderate intensity exercise. Results of studies have been mixed (Gibala et al., 2012), however, HIIT interventions have similar improvements in skeletal muscle metabolic adaptations, cardiovascular fitness, vascular function, glycemic control, and body composition when compared to a much higher volume of MIT (Gibala et al., 2012; Gist, Fedewa, Dishman, & Cureton, 2013; Hazell, Hamilton, Olver, & Lemon, 2014; Macpherson, Hazell, Olver, Paterson, & Lemon, 2011). Since much of the research is mixed and few studies have examined home-based HIIT exercise, this study will make a significant contribution to the literature.

# Study Endpoints/Events/Outcomes

- 1. Primary Endpoint/Event/Outcome: Exercise minutes per week as assessed by an accelerometer for one week at baseline, 6 weeks, and 12 weeks.
  2. Secondary Endpoint(s)/Event(s)/Outcome(s): Self-reported weight and height; psychosocial variables assessed via a questionnaire including social support for exercise, self-efficacy, enjoyment, and outcome expectancies.

# Study Intervention(s)/Investigational Agent(s)

- 1. Description: The intervention will be a 12-week high intensity interval training workout that will consist of home-based exercise sessions prescribed by the exercise counselor. The exercise sessions will be based on exercises the participant can confidentially engage in (regular push-ups vs. knee push-ups vs. wall push-ups). The goal will be to engage in three exercise sessions per week. The participants will receive weekly telephone calls during the first month and bi-weekly calls during months 2 and 3. The exercise counselor will also engage in dialogue that will motivate the participant to exercise. Specifically, the intervention will be based on Self-Determination theory, which is the idea that individuals are more likely to adhere to exercise if they are intrinsically motivated to increase their exercise. According to SDT, intrinsic motivation is enhanced by having a choice and opportunities for self-direction. Although we do not give participants a choice of exercises, we will give them a choice of when they exercise and in what order they complete the exercise sessions. The counseling sessions should be a collaboration between the counselor and participant on how to best integrate exercise into the participant’s daily routine. The counselor will also utilize strategies that will help the individual increase their enjoyment of physical activity.
  2. Drug/Device Handling: NA
  3. Biosafety: NA
  4. Stem Cells: NA

# Procedures Involved

- 1. Study Design: Low active adults will be randomly assigned to either a 12-week home-based high intensity interval training exercise intervention or a wait-list control condition to examine the effect of the intervention on exercise minutes per week.
  2. Study Procedures:

Step 1: Telephone Screening, Consent, and Baseline Questionnaires: In response to an advertisement, potential participants will call, email, or text a study line, at which time they will complete a telephone screening interview with the Research Assistant (RA) to determine eligibility for the study. Participants who are eligible will be given detailed information about the study and the consent form will be reviewed. The RA will schedule a randomization appointment in two weeks. Participants will be told that they may need to cancel the appointment if the online consent and questionnaires are not completed.

1. Complete telephone screening
   1. If not eligible, end interview and thank them for their time
   2. If eligible:
      1. Schedule randomization appointment for 2 weeks
      2. Email link to consent form and questionnaires
      3. Explain ActiGraph procedures. The actigraph is a small device that is attached to the participant’s clothing/belt at the waist and measures physical activity amount and intensity. The actigraph functions as a method of measuring physical activity with the purpose of obtaining objective measurement of the participants’ physical activity.

Step 2: Send ActiGraph: Upon completion of the online consent form and questionnaires, send the ActiGraph with instructions and return envelope.

Step 3: Check on consent and questionnaires

1. If the ActiGraph is not received by the randomization appointment, the participant is to be called to cancel the appointment. The participant should be told that they will be called to reschedule the appointment once the ActiGraph is received.
   1. Once the ActiGraph is received, call the participant to schedule a time or if time permits, complete the randomization appointment at the time of the initial call.
   2. If the ActiGraph has been received, proceed with the scheduled randomization appointment.

Step 4: Baseline Session

1. Participants are randomly assigned to either the exercise or wait-list control condition. The wait-list control condition will have the option of receiving the exercise intervention after the 12 weeks. The exercise intervention will be delivered via phone. Enter randomization date in REDcap.
2. Script: “The next thing we are going to do is to find out which of the two groups you have been randomly assigned to. This is completely random and unfortunately I have no control over which group you are assigned to. Let me access my form here to determine which program you are in.”
3. Exercise: “You have been randomly assigned to the exercise program. This program is designed to help you increase your exercise. I will work with you to make a plan on how you are going to fit exercise into your day. Do you have any questions before we start?”
4. Wait-List Control: “You have been randomly assigned to the wait-list control condition. This means that you will be contacted again in 12 weeks to complete your assessment. After your assessment, you will complete the exercise program if you would like to.”

Step 5: Intervention Sessions

- 1. The participant will be told when exercise should be stopped for safety reasons in order to minimize risk to the participant. See the intervention protocol (a supporting document) for more information. The intervention sessions will be audiotaped for quality control purposes.

**Step 6: 6 week assessment**

1) At 6 weeks, the participant will be prompted to complete the 6 week questionnaire.

Step 7: 12 week assessment

1. At 11 weeks, the participant will be mailed to the ActiGraph, which will be worn for one week starting the day after the last intervention session.
2. At 12 weeks, the participant will be prompted to complete the 12 week questionnaire.
3. The participant will be sent a $50 visa card once the 12 week assessment is complete and the ActiGraph is received.
   1. Study Duration: The study will last 12 weeks for the participant. We anticipate that it will take 12 weeks to enroll all participants and an additional 18 weeks to complete the sessions, 12-week follow-up, and data analysis.
   2. Individually Identifiable Health Information: N/A
   3. Use of radiation: N/A
   4. Use of Center for Magnetic Resonance Research*:* N/A

# Data and Specimen Banking

N/A

# Sharing of Results with Participants

- 1. Results will not be shared with participants.

# Study Population

- 1. Inclusion Criteria: 18 and older; exercising for 90 minutes or less each week; access to the Internet
  2. Exclusion Criteria: Specific exclusion criteria will include a history of coronary heart disease (history of myocardial infarction, symptoms of angina), orthopedic problems that would limit physical activity participation, diabetes, stroke, osteoarthritis, and any other medical condition that may make physical activity unsafe or unwise. Other exclusion criteria will include current or planned pregnancy, psychosis or current suicidal ideation, and psychiatric hospitalization within the last six months.
  3. Screening: Participants will respond to advertisements via text, email, or phone. Participants will complete a telephone screening interview over the telephone to determine eligibility.

# Vulnerable Populations

- 1. Vulnerable Populations:

Children

Pregnant women/Fetuses/Neonates

Prisoners

Adults lacking capacity to consent and/or adults with diminished capacity to consent, including, but not limited to, those with acute medical conditions, psychiatric disorders, neurologic disorders, developmental disorders, and behavioral disorders

Approached for participation in research during a stressful situation such as emergency room setting, childbirth (labor), etc.

Disadvantaged in the distribution of social goods and services such as income, housing, or healthcare

Serious health condition for which there are no satisfactory standard treatments

Fear of negative consequences for not participating in the research (e.g. institutionalization, deportation, disclosure of stigmatizing behavior)

Any other circumstance/dynamic that could increase vulnerability to coercion or exploitation that might influence consent to research or decision to continue in research

Undervalued or disenfranchised social group

Members of the military

Non-English speakers

Those unable to read (illiterate)

Employees of the researcher

Students of the researcher

None of the above

- 1. Additional Safeguards: N/A

Here

# Local Number of Participants

- 1. Local Number of Participants to be Consented: 50

# Local Recruitment Methods

- 1. Recruitment Process: Participants will be recruited via online email advertisements and Craig’s List at the beginning of the study. Interested participants will call, text, or email our study line/email address and participants will be called to complete a telephone screening interview.
  2. Identification of Potential Participants: Participants will self-identify in response to the study advertisements.
  3. Recruitment Materials: Online and email advertisements will be used for the study. The advertisement is included in this IRB submission.
  4. Payment: Participants will received a $50 visa gift card after the 12 week assessment is completed and the ActiGraph is returned.

# Withdrawal of Participants

- 1. Withdrawal Circumstances: Participants will be withdrawn if they demonstrate inappropriate behavior towards the exercise counselor.
  2. Withdrawal Procedures: If the participant states that they would like to withdraw, the research assistant will clarify if they wish to withdraw from both the counseling sessions and follow-up assessments or just the counseling sessions. If the participant wishes to withdraw from both, the participant will not be contacted again. If the participant wishes to withdraw from the counseling sessions only, the research assistant will ask the participant if it is fine for us to contact them for their 6- week and/or 12-week follow-up session. If they say yes, they will be contacted again for this appointment.
  3. Termination Procedures: N/A.

# Risks to Participants

- 1. Foreseeable Risks: There are two risks associated with this study. First, it is possible that a participant would experience a cardiac arrest or some other medical event as a result of exercise. This risk is considered minimal. To address this risk, participants will be given safety instructions that state when they should stop exercising and seek medical help. The second risk is the participant could sustain an injury while exercising. This risk is also considered minimal and participants will be given only exercises that are safe for them. Additionally, to minimize the risk of injury associated with exercise, participants will be screened for orthopedic problems and other medical issues that may make exercise unsafe or unwise. Participants will be advised to use/wear appropriate gear for their chosen form of exercise. If an injury or change in medical status occurs during the study, the intervention will be withdrawn until the participant obtains written clearance from their healthcare provider to continue to engage in exercise.
  2. Reproduction Risks: N/A.
  3. Risks to Others: N/A.

# Potential Benefits to Participants

- 1. Potential Benefits: Exercise is related to numerous health benefits including reduced risk of heart disease, stroke, some types of cancer, and obesity. If the participant adheres to the exercise protocol, they could experience any of these benefits.

# Statistical Considerations

- 1. Data Analysis Plan: Between groups ANOVA tests will be used to determine if there are significant differences between the two groups on exercise minutes per week, weight, and the psychosocial variables.
  2. Power Analysis: This is considered a pilot study and therefore, a power analysis has not been completed.
  3. Statistical Analysis: See 15.1 above.
  4. Data Integrity: When completing the online questionnaires, participants will be prompted if they miss an item. They will not be allowed to submit their questionnaire until they have completed all items. The research assistant will also look over the ActiGraph data to make sure it has been worn each day and exclude any days it appears it has not been worn.

# Confidentiality

- 1. Data Security: As dictated by the study protocol, participants will complete questionnaires online. Information obtained from these questionnaires will be housed on a highly secured server, which will include a firewall, password protection, and several other precautionary mechanisms. Identifying information will not be included with the questionnaires. Participants will be assigned a study ID upon entering the study and only the study identification number will be included with the questionnaires. All data we collect will remain confidential. The information that matches the participant’s name with the participant’s study identification number, telephone numbers, and mailing addresses will be kept in a separate file from the questionnaire data. The ActiGraph data will be stored in files that include the participant’s study ID only.

# Provisions to Monitor the Data to Ensure the Safety of Participants

- 1. Data Integrity Monitoring. This study is considered a minimal risk study and therefore, will not include a data safety monitor or data safety monitoring board. However, there will be several steps taken to ensure the integrity of the data. Specifically, in order to gain access to the study data, the computerized data entry system will require a login and password. We will apply range rules and validation to the actual entry fields, where appropriate. Since the questionnaires are online, the data will be available directly to the research associate. The research associate will also be responsible for downloading the ActiGraph data. The research associate will make the data available to the Principal Investigator and other staff associated with the project once the study is completed, and all data have been collected, entered, and passed the audit procedures. The Principal Investigator will be the only person who can give permission for release of the aggregated data. Confidential information will not be released without the express written consent of the study participants. The original data file will stay in its original state.
  2. Data Safety Monitoring. All adverse events will be promptly reported to the IRB. In the proposal, one potential adverse event is injury due to participation in exercise. Participants will be instructed to call their healthcare provider and the exercise counselor for the current study if an injury were to occur. Additionally, to be proactive, participants will be asked during the telephone session if they have experienced any injuries.

# Provisions to Protect the Privacy Interests of Participants

- 1. Protecting Privacy: Data on individual participants will not be released. The data will only be presented in aggregate form and participants’ identifying information will not be shared outside of the research personnel who need to have direct contact with the participants.
  2. Access to Participants*:* N/A. This study does not involve accessing medical records.

# Compensation for Research-Related Injury

- 1. Compensation for Research-Related Injury: N/A. This study does not involve greater than minimal risk.
  2. Contract Language: N/A

# Consent Process

- 1. Consent Process (when consent will be obtained): The research associate will describe the consent form to the participants during the telephone screening interview. Immediately following the interview, participants will be sent an online consent form. The participants will be told to call the study line if they have any questions when completing the consent form. Participants will sign the consent form electronically which will then be submitted directly to the research associate. The consent form is attached to this application.
  2. Waiver or Alteration of Consent Process (when consent will not be obtained): N/A
  3. Non-English Speaking Participants: N/A
  4. Participants Who Are Not Yet Adults (infants, children, teenagers under 18 years of age): N/A
  5. Cognitively Impaired Adults, or adults with fluctuating or diminished capacity to consent: N/A
  6. Adults Unable to Consent: N/A

# Setting

- 1. Research Sites: The research team will be housed in the School of Kinesiology at the University of Minnesota but all research activities will take place in the participant’s home on their own. The exercise counseling will occur over the telephone with specific instructions regarding how to complete the exercise sessions at home.
  2. International Research: N/A

# Multi-Site Research: N/A

# Resources Available

- 1. Resources Available: Based on the number of potential participants that are advertisements will reach, we believe that our recruitment target is feasible. We anticipate completing the entire research project in 6-9 months. The research activities are conducted out of an office that houses 2-3 research assistants, which have access to email and phones needed for the study. The computers are equipped with software that allows for downloading and analyzing the ActiGraph data. The PI is a licensed psychologist and therefore, if there are any psychological resources needed, she will provide these resources. All research assistants will be adequately trained on the research protocol. The research coordinator for this project has conducted several randomized trials and is familiar with the procedures involved in this type of project. She will oversee the research assistants on this projects to ensure that the protocol described in this application is adhered to. Additionally, as mentioned above, the exercise counseling sessions will be audiotaped. Ten percent of the audiotapes will be listened to by the PI on this study to ensure that fidelity to the treatment protocol is maintained. If the study protocol is not followed, the research assistants will be re-trained on the study procedures and the next five sessions will be audiotaped and listened to. This procedure will continue until the protocol is being followed. If the research assistant is not able to adhere to the protocol, they will be terminated.

# References

Burgomaster, K. A., Howarth, K. R., Phillips, S. M., Rakobowchuk, M., MacDonald, M. J., McGee, S. L., & Gibala, M. J. (2008). Similar metabolic adaptations during exercise after low volume sprint interval and traditional endurance training in humans. *The Journal of Physiology*, *586*(1), 151-160.

Gibala, M. J., Little, J. P., Van Essen, M., Wilkin, G. P., Burgomaster, K. A., Safdar, A., ... & Tarnopolsky, M. A. (2006). Short‐term sprint interval versus traditional endurance training: similar initial adaptations in human skeletal muscle and exercise performance. *The Journal of Physiology*, 575(3), 901-911.

Gibala, M.J, Little, J.P., Macdonald, M.J., & Hawley, J.A. (2012). Physiological adaptations to low-volume, high-intensity interval training in health and disease. *Journal of Physiology*, 590(Pt 5), 1077–84.

Gist, N. H., Fedewa, M. V., Dishman, R. K., & Cureton, K. J. (2014). Sprint interval training effects on aerobic capacity: a systematic review and meta-analysis. *Sports Medicine*, *44*(2), 269-279.

Hazell, T.J., Hamilton, C.D., Olver, T.D., & Lemon, P.W. (2014). Running sprint interval training induces fat loss in women. *Applied Physiology, Nutrition, and Metabolism,* 39(8), 944–50.

Macpherson, R.E., Hazell, T.J., Olver, T.D., Paterson, D.H., & Lemon, P.W. (2011). Run sprint interval training improves aerobic performance but not maximal cardiac output. *Medicine & Science in Sports & Exercise*, 43(1), 115–22.
